# Epigenetics in Social Insects: A New Direction for Understanding the Evolution of Castes

**DOI:** 10.1155/2012/609810

**Published:** 2012-03-28

**Authors:** Susan A. Weiner, Amy L. Toth

**Affiliations:** ^1^Department of Ecology, Evolution, and Organismal Biology, Iowa State University, Ames, IA 50014, USA; ^2^Department of Entomology, Iowa State University, Ames, IA 50014, USA

## Abstract

Epigenetic modifications to DNA, such as DNA methylation, can expand a genome's regulatory flexibility, and thus may contribute to the evolution of phenotypic plasticity. Recent work has demonstrated the importance of DNA methylation in alternative queen and worker “castes” in social insects, particularly honeybees. Social insects are an excellent system for addressing questions about epigenetics and evolution because: (1) they have dramatic caste polyphenisms that appear to be tied to differential methylation, (2) DNA methylation is widespread in various groups of social insects, and (3) there are intriguing connections between the social environment and DNA methylation in many species, from insects to mammals. In this article, we review research on honeybees, and, when available, other social insects, on DNA methylation and queen and worker caste differences. We outline a conceptual framework for the effects of methylation on caste determination in honeybees that may help guide studies of epigenetic regulation in other polyphenic taxa. Finally, we suggest future paths of study for social insect epigenetic research, including the importance of comparative studies of DNA methylation on a broader range of species, and highlight some key unanswered mechanistic questions about how DNA methylation affects gene regulation.

## 1. Introduction

**Figure 1:**
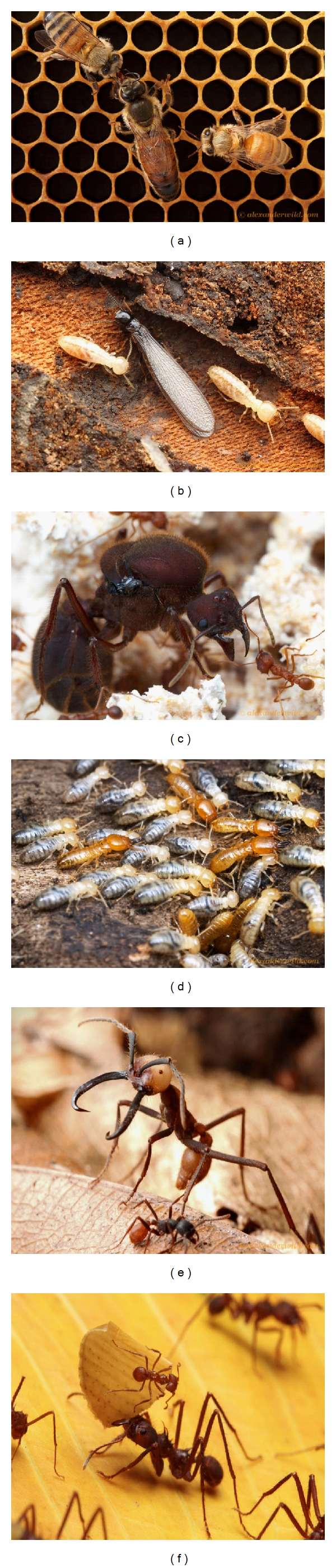
Examples of striking phenotypic plasticity between castes in the social insects. (a) Honey bee queen (center) and workers. (b) A winged reproductive termite *Reticulitermes flavipes* (center) and nonreproductive workers. (c) Queen leafcutter ant *Atta texana* (center) and a daughter worker (left). (d) Soldiers (with larger mandibles) and workers of the termite *Prorhinotermes inopinatus*. (e) An army ant *Eciton burcelli *soldier (center) and minor worker (bottom). (f) Major and minor workers of the leafcutter ant *Atta cephalotes*. All photos used by permission from Alex Wild.

Phenotypic plasticity is an important biological phenomenon that allows organisms with same genotype to respond adaptively to variable biotic and abiotic environments. There are several molecular mechanisms that can contribute to genomic flexibility and thus phenotypic plasticity, including transcriptional regulation, posttranscriptional modification, alternative splicing, and epigenetic modifications of DNA (reviewed in [1]). In this paper, we explore the potential role of epigenetic modifications in phenotypic plasticity in social insects in the order Hymenoptera (bees, ants, and wasps), a group of animals that exhibit many remarkable forms of morphological and behavioral plasticity [2]. Phenotypic polymorphism has arisen many times in different insect lineages [3] and not always among eusocial insects. Other well-studied examples of extreme phenotypic plasticity in insects include pea aphids with winged and wingless morphs, as well as sexual and asexual generations (reviewed in [4]), horned and hornless morphs in dung beetles [5], and phase differences in migratory locusts [6]. Studies of insects, and especially social insects, are providing intriguing new insights into the relevance of epigenetic modifications of DNA to the evolution of phenotypic plasticity [7, 8]. Eusocial insects provide some of the most dramatic examples of polyphenism found in any organism (Figure 1). 

The colonies of eusocial insects can be highly complex, organized systems, sometimes containing tens of thousands or even millions of individuals [2]. In these colonies, despite the vast number of individuals, only a small percentage of individuals ever reproduce. In fact, in highly eusocial organisms such as honeybees, the workers have lost the ability to mate. The evolution of sterile workers has been a major evolutionary puzzle since Darwin [9]. One aspect that deeply concerned Darwin was that sterile female workers could be morphologically quite different from queens. Queens are generally larger, longer lived, and have large ovaries and a high reproductive output. In some species they can have vastly different body proportions and morphological characters compared to workers (Figure 1). How could such different phenotypes (castes) evolve if workers leave no descendants upon which natural selection can act? In some ant species, these caste systems are even more striking, with the presence of two or more types of morphologically distinct workers (e.g, specialized soldiers, major, and minor worker castes, Figure 1, [10]).

 The extreme phenotypic plasticity of social insect castes has become even more compelling with the knowledge that, in most species, queen and worker caste differences are environmentally, not genetically, determined. With some notable exceptions, such as some genera of ants (e.g., [11–15]), in most social insects, there are no heritable genetic differences that dictate which individuals become queens and which become workers, nor among different morphological castes of workers [16]. Thus, the genomes of many social insects possess remarkable phenotypic flexibility, which is exquisitely sensitive to the abiotic and social environment (reviewed in [17]). Depending on the species and level of sociality, caste differences can range from being completely behavioral and physiological (e.g., in *Polistes* paper wasps, [18]) through showing dramatically different alternate morphological phenotypes or polyphenisms (e.g., honey bees, Figure 1, [19]).

Work in rats and other mammals has uncovered that epigenetic modifications of DNA are important for mediating the effect of the early social (maternal) environment on adult phenotype (reviewed in [7, 20]). This work led to the suggestion that social modulation of the genome, and the resulting adult plasticity, may rely heavily on epigenetic effects [20]. This suggestion is made even more intriguing by the discovery that epigenetic effects are also important for caste determination in highly social honey bees [21–23] and likely in other social insect species [24]. In this paper, we summarize progress on epigenetics in social insects and compare this to work in other animals, in order to broaden the perspective on social insect studies. We also synthesize existing data into a conceptual framework of how epigenetic modifications of DNA may affect queen-worker caste phenotypes in social insects. Finally, we use this background to suggest what could be done to move the emerging field of social insect epigenetics forward. 

## 2. Epigenetic Modifications of DNA

To facilitate our discussion of the importance of epigenetic modifications to social behavior in insects, we must first clarify what we mean by epigenetics. The term “epigenetics” has been used in a wide variety of contexts, to describe both organism-level and molecular-level phenomena [7]. Here, we refer specifically to chemical modifications to DNA that do not change the DNA sequence [7]. These modifications can be tissue specific or consistent throughout different cell types [25]. Epigenetic modifications can be made to DNA or to the histones on which DNA is stored [20]. They can even be transmitted from parents to offspring, so they can be stable over many cell divisions, though they can also be reversible (reviewed in [7]). Modifications present in the parental genome may be passed on, or new modifications may be made in the DNA of the gametes [7, 26]. This can lead to imprinting, in which paternal and maternal genes are differentially expressed [27].

A rough analogy can be made that the DNA sequence is like a written language with no spaces, capitalization, or punctuation. In other words, it contains the information to produce an organism, but that information cannot be properly decoded and understood in its raw form. Epigenetic modifications can be viewed as embellishments to the DNA language, providing punctuation that allows strings of nucleotides to be read and contain meaningful information. On a biochemical level, these modifications can help define the level at which genes are expressed (reviewed in [28]) and may also influence alternative splicing [23, 29].

 Epigenetic DNA modifications can take several forms. Methyl groups can be added directly to nucleotides in a process called DNA methylation [30]. Primarily methylation occurs at the cytosines in CG dinucleotides, but methylation can occur on other cytosines or even other nucleotides [31]. In addition, modifications can be made to the histones around which DNA is packaged [20]. These modifications include methylation, acetylation, and ubiquitination [32]. All these different modifications have the potential to affect transcription via changes in chromatin structure and/or gene splicing patterns [20, 23, 32]. Most of the current literature, particularly in social insects, has focused on DNA methylation, so this paper will also focus on DNA methylation. However, histone acetylation is strongly negatively correlated with DNA methylation, and the two may be maintained in a dynamic equilibrium [20]; thus, it is important to keep in mind that other types of epigenetic modifications may have equally important effects on gene regulation.

 DNA methylation appears to be an ancestral trait in eukaryotes but may serve different purposes in different taxa [33]. In plants and vertebrates, DNA methylation is important for the suppression of transposable elements [33]. Transposable elements are DNA sequences that can move themselves from one location to another in the genome, either by copying themselves or by cutting out of one region and reattaching elsewhere. In vertebrates, regions-containing transposable elements are heavily methylated, which both suppresses their expression and inactivates them over time by increasing the rate of mutation [34]. These elements tend to be more common in plants and vertebrates, although invertebrates are more subject to the effects of transposons than mammals, suggesting that one of the benefits of methylation is as a defense against transposons [35]. Gene body methylation is also common in plants and animals (but less so in fungi) [33]. In invertebrate animals, in particular, most methylation occurs within gene bodies [33]. Methylation can also occur at promoters or other noncoding regions, particularly in vertebrates and plants. When promoter regions are methylated, the expression of the gene or region is generally silenced [33].

Although DNA methylation has been associated with silencing of gene expression in vertebrates, more recent studies in insects suggest that gene body methylation is highest in genes with intermediate expression and in genes that are ubiquitously expressed in different tissues [22, 33, 36]. Differential methylation of a gene between different tissue types, social roles, or life stages may have important effects on gene expression [20, 21, 23, 33, 37]. In vertebrates, socially mediated methylation is known in promoter regions [20], but in insects, all evidence thus far suggests that social effects on methylation, and indeed nearly all methylation, occurs within gene bodies [33]. Methylation within genes may regulate splicing by an as-of-yet poorly understood mechanism [23, 38, 39].

The enzymatic addition of methyl groups to nucleotides involves several DNA methyltransferases (DNMTs). Most organisms with a fully functional DNA methylation system have at least one copy of each of DNMT1, DNMT2, and DNMT3. DNMT1 maintains methyl tags, while DNMT3 is involved in *de novo* methylation. DNMT2 is not considered a true DNA methyltransferase and may be involved in methylation of tRNAs [40, 41]. Despite the many important functions of DNA methylation, some organisms do without a complete set of methylation enzymes in their genomes and have little or no methylation in their DNA [33]. For example, *Drosophila* does not possess DNMT1 or DNMT3 orthologs (reviewed in [33]). Therefore*, Drosophila* has very little methylation in its genome (reviewed in [41]) although a low level is present in embryonic stages [42]. Because of early studies with *Drosophila*, it was initially thought that DNA methylation was not important in insects (reviewed in [41]). However, recent work has revealed evidence of DNA methylation in several insects, including all Hymenoptera and some Orthoptera (crickets), Hemiptera (aphids), and Lepidoptera (moths) ([24, 33, 43], reviewed in [44]). DNA methylation is inferred to occur in all eusocial insects thus far examined [24, 45, 46]. Other insects with phenotypic polymorphism such as aphids have also been demonstrated to possess moderate levels of genome-wide methylation [47, 48].

There are many open questions relating to our understanding of how DNA methylation affects phenotype, and the social insects are a promising new model with which to better understand these questions. (1) Are epigenetic modifications of DNA a key mechanism in the evolution of extreme phenotypic plasticity [8]? (2) Did epigenetic effects facilitate the evolution of division of labor and eusociality? (3) What is the raison d'etre of epigenetic DNA modifications, and can the study of this theme in social insects help shed light on this question?

## 3. Connections between Epigenetics and Sociality

Because of their potential to be passed between generations, epigenetic changes to DNA have been of great interest as mediators of intragenomic conflict. Observations have long suggested that genes from maternal and paternal genomes (matrigenes and patrigenes) may have opposing effects on offspring phenotypes, such as the amount of resources offspring take from their mothers [27]. Paternally imprinted genes tend to cause offspring to take more resources to maximize their own fitness, while maternally imprinted genes tend to decrease the amount of resources taken to allow the mother to spread her investment over more offspring.

In a haplodiploid system such as the eusocial Hymenoptera (ants, bees, and wasps), it has been suggested matrigenes and patrigenes will be in further conflict over the treatment of social partners and offspring to which they are differentially related [26]. The haplodiploid genetic system of hymenopteran insects, in which females are diploid and males are haploid, results in “supersister” relationships in which sisters with the same father are on average 75% related [49]. Queller [26] predicted that genes promoting reproductive cooperation among closely related (e.g., supersister) females founding a nest together (such as in paper wasps in the genus *Polistes*) would be paternally imprinted (because patrigenes will be 100% shared whereas matrigenes only 50% shared). These and many other predictions related to imprinting in social insects still await experimental verification. Some of these questions could potentially be addressed by looking specifically at germline methylation. However, even in mammalian systems, in which imprinting has been best studied, imprinting through methylation is relatively uncommon and its mechanisms are still poorly understood [50].

Research on mammals has found that DNA methylation can be very important in mediating the effects of early life nutrition and social circumstances on phenotype [7, 20, 37, 51]. For example, rat pups that are cared for by more attentive mothers (mothers that perform more grooming and arched-back nursing behaviors) are less reactive to stress later in life [37]. This change is mediated by enhanced methylation of the exon 1_7_ promoter of the glucocorticoid receptor in pups cared for by less attentive mothers [37]. Feeding adults methionine increased methylation of this exon and caused adults that were cared for by attentive mothers to have behavioral stress responses typical of rats receiving poor maternal care, indicating that methylation is changeable even in adult life and that methylation levels are directly linked to behavioral differences [51]. This work, in addition to other studies in mammals (reviewed in [52]), suggests that DNA methylation can be a key mechanistic link between the genome and the maternal and social environment [20].

Maternal effects (or similar effects mediated by workers) are very important in caste determination in social insects [26, 53, 54]. It is well known that brood-caregiver interactions (whether between mothers and offspring or workers and alloparental brood) are essential to caste differences [53, 54]. This can occur via differential feeding or nourishment [55], pheromonal signaling [56] or even vibrational cues [57]. Thus, there are fascinating (and heretofore unexplored) parallels between the potential effects of the maternal environment in mammals and brood care effects in social insects. A rough analogy between mammalian maternal effects and social/nutritional effects on caste determination in social insects suggests great potential for the role of DNA methylation in insect social organization [45].

## 4. Evidence for DNA Methylation in Social Insects

Evidence to date suggests important and widespread roles for DNA methylation in the Hymenoptera. The honey bee genome revealed that honeybees possess a complete set of DNA methyltransferases (two copies of DNMT1, and one each of DNMT2 and DNMT3) and DNA methylation has been experimentally verified in several studies [41, 45, 46]. Subsequently, a full complement of DNMTs was discovered in the solitary parasitoid jewel wasps, *Nasonia vitripennis* and two closely related species [58], as well as in 7 recently sequenced ant genomes ([59–64], reviewed in [44]) and in the paper wasp *Polistes dominulus* (A. L. Toth, unpublished data).

While the honey bee and *Nasonia* possess multiple copies of DNMT1 (three in *Nasonia* and two in honey bees), all sequenced ant genomes show evidence for only one DNMT1 (reviewed in [44]). This suggests the number of DNMT1 genes is evolutionarily labile within the Hymenoptera; however, further studies on additional solitary and social taxa are needed to understand this pattern of apparent expansion and contraction of DNMT1 genes. Based on rough estimates using methylation-sensitive restriction enzyme assays, relatively high levels of methylation (similar to or higher than that in the honeybee) have been estimated in the paper wasp *Polistes dominulus*, the carpenter ant *Camponotus festinatus*, the advanced eusocial wasp *Polybia sericea, *and the yellowjacket *Vespula pennsylvanicus* [24], as well as the harvester ant *Pogonomyrmex barbatus *[61]. Somewhat lower levels have been estimated in several other social Hymenoptera, including several advanced eusocial species as well as a small set of more primitively eusocial species [24]. Subsequent studies also experimentally confirmed the presence of DNA methylation in the genomes of the fire ant *Solenopsis invicta *[64] as well as the jumping ant *Harpegnathos saltator* and the carpenter ant *Camponotus floridanus *[59]. The latter study suggested lower DNA methylation levels may be associated with the more primitively eusocial lifestyle of *H. saltator* compared to *C. floridanus* [59].

Some insects have little to no DNA methylation (e.g., the flour beetle *Tribolium castaneum,* the flies *Anopheles gambiae, *and *Drosophila melanogaster*, [22], reviewed in [44]). Recently, however, it has come to light that many other invertebrates possess a full complement of DNA methylation enzymes and/or show genome wide levels of DNA methylation that are comparable to those of the Hymenoptera (*Daphnia *water flea: [65], stick insect: [66], crickets: [67], cabbage moth: [43], silkworm: [68], aphids: [47, 48, 69, 70], and human body louse: [71], also reviewed in [44]). This suggests that, while methylation may be important for eusociality, it is by no means unique to social taxa among insects. This indicates that DNA methylation, while not essential to all insects, may play distinct and important roles in certain insect groups. We do not yet know of the presence, nor the extent of divergence of methylation systems in many lineages of insects; thus there is a great deal still to be learned about what factors drive the maintenance or loss of DNA methylation machinery in insects.

## 5. DNA Methylation and Caste Determination

After the discovery of a functional DNA methylation system with the sequencing of the honey bee genome [45], there has been a flurry of research to better understand the significance of DNA methylation in honeybees and, in particular, how methylation affects caste determination. Kucharski and colleagues [21] inhibited the expression *of dnmt3*, the *de novo* DNA methyltransferase, in worker larvae, which typically have elevated *dnmt3* expression compared to queen larvae [72]. They demonstrated that *dnmt3 *knockdown caused demethylation of a biomarker gene, *dynactin p62*. Typically, *dynactin p62* is more highly methylated in worker honeybees than in queens, and queen larvae show higher expression of *dynactin p62*, though its role in caste determination is not known [21]. After *dmnt3 *knockdown, emerging adults showed queen-like traits, both phenotypically (larger size, larger ovaries, and queen-like morphological traits) and in their methylation patterns. These data strongly suggested DNA methylation plays a direct causal role in honey bee caste determination, and this striking finding led to a series of studies, both experimental and computational, aimed at characterizing the “methylome” or complete set of methylated sites, in the honey bee genome.

 In order to estimate DNA methylation levels in sequenced genomes, researchers have used bioinformatic approaches, focused on the CpG dinucleotide content of genes [22, 36]. Methylation primarily occurs on the cytosines of CpG dinucleotides. Methylated cytosines are more prone to mutation, and, therefore, regions that are consistently highly methylated will, over time, become CpG depleted [22]. The fruit fly *Drosophila melanogaster*, the mosquito *Anopheles gambiae*, and the flour beetle *Tribolium castaneum* (all of which have little to no DNA methylation) have a unimodal distribution of CpG richness [22]. Honeybees, like several other organisms with substantial DNA methylation, have a bimodal distribution of CpG richness in their genes, indicating that some genes are highly methylated (leading to CpG depletion) and some genes are nonmethylated or weakly methylated (allowing for the maintenance of CpG rich DNA) [22]. The solitary parasitoid wasp *Nasonia vitripennis* also shows a bimodal distribution of CpG richness, which is more pronounced in introns [61]. However, more recent evidence suggests the classic bimodal pattern may not always be present in insect species with functional methylation systems. In two ants, *Pogonomyrmex barbatus* and *Linepithema humile,* despite the presence of a full complement of DNMTs and experimental evidence of DNA methylation, there is no evidence of bimodality in CpG content in exons nor in introns [61, 62].

 The aforementioned data on CpG composition in honey bees were subsequently used to examine connections between DNA methylation and gene expression. Lists of “predicted methylated genes” in the honey bee genome were compared to global gene expression data (using microarrays). These analyses found that genes predicted to be most heavily methylated in honeybees were ubiquitously expressed “housekeeping genes” involved in basic biological processes such as cell communication, development, cell adhesion, and signal transduction [22, 36].

However, because CpG content measurements are based on mutational changes, they only reflect methylation patterns of genes that are methylated in the germ line, as somatic mutations will not be passed on to the next generation nor accumulate over time [22]. Such a limitation could potentially be more serious in the honeybee. Since workers rarely reproduce, genes that are methylated in workers but not in queens or males would not be expected to show substantial CpG depletion. Thus, this method may not pick up key differences in methylation between castes, nor in genes that are methylated in specific tissues, but not in the germline. Nonetheless, to date there is good agreement between CpG predictions of methylation status and the actual presence of DNA methylation [36], supporting the use of this metric as a proxy for methylation status.

 Experimental approaches have uncovered evidence of differential methylation of particular genes in queens and workers [21, 23]. Foret and colleagues [36] confirmed their bioinformatic assessment of methylation levels of several genes from CpG content estimates with bisulfite sequencing. Bisulfite sequencing involves treating DNA with bisulfite, which converts unmethylated cytosines into uracils, but leaves methylated cytosines. By treating DNA with bisulfite and then comparing the sequences to untreated DNA, methylated cytosines can be identified. This method has been used to demonstrate differential methylation in several genes, including *dynactin p62* [21, 72] and *hexamerin 110* [73]. Differential methylation of *dynactin* has been demonstrated to correlate with queen-like and worker-like traits, even in intercastes when rearing changes are made after the critical period [72]. However, to date, there have been no demonstrated causal roles for any known differentially methylated gene, including *dynactin p62* and *hexamerin 110*. These are clearly areas that are ripe for future study.

In honeybees, evidence to date is unclear on how differential methylation is relevant to caste-specific differential gene expression; relatively few differentially methylated genes have actually turned out to be differentially expressed between castes [21–23, 44]. However, new evidence from both honey bees [23] and mammalian cells [39] suggests differential methylation may be important for alternative splicing. Based on studies in human lymphoma cell lines, Shukla and colleagues [39] proposed a potential mechanism linking gene body methylation with splicing. Their data suggest that CTCF, a DNA-binding protein that promotes exon inclusion during transcription, is inhibited by gene body methylation. In this way, DNA methylation may affect the frequency of transcription of certain exons.

In honeybees, there is also evidence for a connection between DNA methylation and alternative splicing. GB18602 is a gene that has two splice variants, one that is found in both queens and workers and one that is significantly upregulated in queens [23]. GB18602 is also differentially methylated in the brains of queens and workers, particularly around the areas of alternate splicing, suggesting that the differential methylation is relevant to the splicing [23]. Using bisulfite sequencing on a genomic scale, Lyko et al. [23] identified hundreds of putative differentially methylated genes encoding highly conserved proteins involved in core cell functions. In the brains of adult queens and workers, 550 differentially methylated genes were found, including genes involved in metabolism, RNA synthesis, nucleic acid binding, and signal transduction [23].

## 6. Conceptual Framework

 In the paragraphs that follow, we have synthesized existing information from honey bees into a conceptual framework to describe the potential role of DNA methylation in caste determination in social insects. First, we suggest that DNA methylation in social insects can be divided into two types: consistent and differential (Table 1). Both types of methylation are primarily found in gene bodies and particularly exons [33].

Consistent methylation describes sites that are equally likely to be methylated across different castes and tissues. We predict that these genes will tend to have deeply conserved methylation patterns that are shared across a wide variety of insect taxa, for example, pea aphids and honey bees [47]. The functions, as well as the sequences of consistently methylated genes, appear to be especially well conserved over hundreds of millions of years of insect evolution [47]. Evidence from honey bees suggests consistently methylated genes are consitutively expressed across tissues and castes and are involved in core cell functions [22]. Genes that are consistently methylated in the germline should be accompanied by decreased CpG content due to mutation of methylated cytosines over time [22]. (Note that low CpG content may potentially identify both genes that are truly consistently methylated, as well as genes that are differentially methylated but more highly methylated in the germ line of queens).

Differential methylation describes sites that are more likely to be methylated in certain tissues or castes. Differentially methylated genes are predicted to be more variable in their expression and/or splicing patterns in space, time, and across individuals [23]; however, at this time there is limited empirical data on how differential methylation actually affects gene regulation in social insects. Areas with higher methylation in workers and in nongermline tissue are less likely to accumulate CpG-depleting mutations over time [22, 23]. Evidence to date suggests that differentially methylated genes in honey bees tend to have higher CpG content than genes that are consistently methylated, though they still show some evidence of moderate CpG depletion [23].

Differential methylation has been demonstrated to be involved in caste determination in honeybees [21], although the exact mechanism by which differential methylation is translated into differential gene regulation is not yet clear. Caste in honeybees is also known to be controlled by environmental factors, especially larval nutrition, which have downstream effects on hormonal signaling (e.g., juvenile hormone), gene expression, and developmental fate [55]. A recent study has also demonstrated the importance of a dietary factor, the peptide royalactin in royal jelly, that may stimulate growth factor signaling pathways, leading to queen development [74]. The effect of nutrition on methylation in mammals has been well documented, particularly in transgenerational metabolic syndromes (reviewed in [75]); thus it is intriguing to postulate a similar role in social insects. Evidence to date suggests the effects of diet on caste phenotype can be mediated by methylation of particular genes [21]. This differential methylation could potentially affect both expression and splicing, both of which can contribute to the expression of alternative phenotypes through the activation of different gene networks.

**Figure 2:**
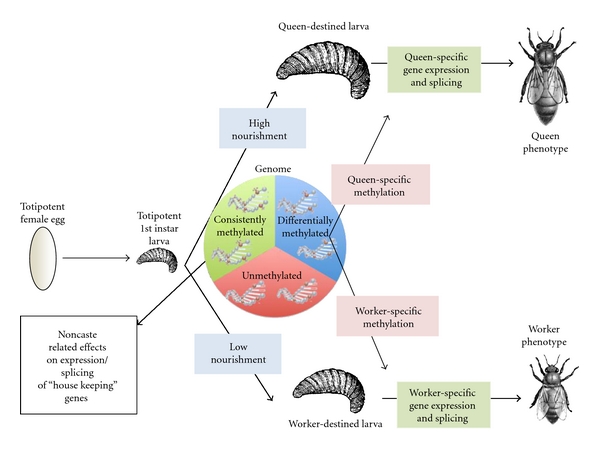
Schematic diagram describing the role of DNA methylation in caste determination in honey bees. Each female egg begins in a totipotent state, which lasts through early larval instars, that can potentially develop into either a queen or worker. Differential nourishment, in the form of royal jelly in the case of queens and lower-quality/quantity food in the case of workers, differentially affects the genomes of queen-and worker-destined larvae. The genome can be roughly divided into unmethylated DNA, consistently methylated DNA, and differentially methylated DNA. Differential methylation can potentially affect the downstream levels of expression and splicing patterns of many genes related to growth, metabolism, and development, leading to alternative queen and worker phenotypes.

In our conceptual framework (Figure 2), we propose that dietary differences lead to differential methylation. This, in turn, leads to alternative splicing and possibly caste-biased expression, which leads to caste-biased phenotypes, such as restricted ovarian development in workers or larger body size and longer lifespan in queens (Figure 2). Numerous studies have already begun to identify specific genes and pathways associated with queen and worker caste determination in honey bees (reviewed in [17]). These include significant changes in gene expression of storage proteins [76], mitochondrial enzymes [77], lipid metabolism enzymes [78], insulin pathway genes [79], heat shock proteins [78], and growth factors [74]. It remains to be seen whether differential methylation directly affects the expression and/or splicing patterns of these genes, or whether they are downstream effectors of other differentially methylated genes.

The purpose of consistent methylation is less well understood. Methylation in honeybees occurs primarily in exons, and methylated cytosines have a higher mutation rate, which should incur a cost to maintaining high levels of methylation. The presence of consistent methylation that is conserved over millions of years of insect evolution [47] suggests that consistent methylation is serving some important purpose, or it would be selected against; indeed DNA methylation has been lost in some insects [33]. However, despite the higher mutation rates of methylated genes, many methylated genes are especially highly conserved on the protein level, suggesting strong selection against sequence divergence in these genes [23]. One possibility is that methylation of certain classes of genes may repress potentially damaging alternative transcription patterns; this may be especially important in “housekeeping” genes that are ubiquitously expressed across many tissue types [44]. Nonetheless, it is also possible that consistent methylation could be a nonadaptive side effect of the evolutionary maintenance of DNA methylation systems for differential methylation.

## 7. Where Do We Go from Here?

There is still a great deal still to be learned going forward in the study of DNA methylation in social insects. Important groundwork has been laid in *Apis mellifera*, but we do not yet know whether DNA methylation is relevant to caste differences in other social species. We suggest it will be particularly illuminating to take a comparative perspective on the study of DNA methylation and castes in Hymenoptera, as this group represents at least 11 different origins of sociality and has species with various different levels of sociality, from facultatively social to advanced eusocial, and even some lineages in which sociality has been lost or obligate social parasitism has evolved. In each of these cases, comparisons to what is currently known about honey bees could provide many useful and interesting answers to a long list of open questions relating to epigenetics and the evolution of sociality. Below, we provide a few provocative examples.

### 7.1. DNA Methylation and Caste Determination

#### 7.1.1. Is DNA Methylation Important in Social Organization during the Early Stages of Social Evolution, or Is It more of a Feature of Highly Derived Social Systems Such as Honey Bees?

Data thus far suggest some primitively social lineages, such as the paper wasps *Polistes dominulus* have even higher DNA methylation levels than the advanced eusocial honey bees [24]. In primitively eusocial species, queen and worker castes are phenotypically very similar, and adults can switch between castes, but each individual actually retains *greater* phenotypic plasticity in its behavior and physiology throughout its lifetime than in an advanced eusocial species. Thus, it is possible that DNA methylation could be as important or even more important in mediating phenotypic plasticity during the early stages of eusocial evolution.

#### 7.1.2. Does Having a Functional DNA Methylation System in Place Predispose or Allow a Lineage to Evolve a Broader Range of Phenotypic Plasticity?

Eusocial Hymenoptera, and their nonsocial kin within the aculeate (stinging Hymenoptera) lineage, evolved from parasitoid ancestors. We know that members of at least one parasitoid Hymenopteran lineage, the jewel wasps in the genus *Nasonia*, do possess a fully functional methylation system suggesting such a system existed in the solitary ancestors of social Hymenoptera. This suggests that the solitary ancestors of social Hymenoptera already possessed a fully functional DNA methylation system. Could the existence of a DNA methylation system have provided a baseline level of genomic plasticity that allowed for or facilitated the evolution of different castes? Regev et al. [80] suggested that within invertebrates, higher DNA methylation was associated with higher rates of cell turnover, and perhaps developmental complexity and/or flexibility. Gaining a better understanding of the association between developmental plasticity and DNA methylation could begin to provide some hints about the adaptive advantages conferred by evolutionary maintenance of DNA methylation machinery.

#### 7.1.3. What Happens to DNA Methylation Systems When Sociality Is Lost, or When the Queen or Worker Caste Is Lost, during Evolution?

If DNA methylation is maintaining phenotypic plasticity in eusocial species, we may expect relaxed selection or evolutionary changes in DNA methylation patterns and DNMT enzymes in species in which sociality is lost. For example, It would be informative to examine DNA methylation systems in species where caste polyphenism is lost or reduced, e.g. in halictid (sweat) bees in which there have been reversions to solitary behavior [81], during the evolution of queenless or workerless social parasites (as found in several bee, ant, and wasp lineages) [82], or in cases where morphological caste differences have been secondarily reduced as in the swarm founding wasps [83]. If caste flexibility is lost, is selection for the maintenance of DNA methylation systems also relaxed?

#### 7.1.4. Does DNA Methylation Play a Role in Caste Differentiation in Multiple, Independent Origins of Sociality, and If so, Are the Same Genes and/or Pathways Methylated in Each Origin, or Are These Largely Lineage Specific?

Functional DNA methylation systems are now inferred to be present in numerous species of social bees, ants, and wasps [24, 59–64]. Based on gene expression studies in a wide variety of social Hymenoptera (reviewed in [17]), it appears that many of the same genes and pathways, especially those involved in metabolism, nutrient signaling, and hormone signaling, are involved in caste determination across a wide variety of species as well. If caste-related expression differences are convergent, and methylation is involved in caste differences in multiple lineages, are differential methylation patterns associated with caste differences also convergent across social insect taxa?

#### 7.1.5. What Role Does Methylation Play in Nonhymenopteran Eusocial Systems?

Thus far, there is no published work on DNA methylation in termites or other nonhymenopteran social arthropods with castes such as aphids, thrips, or snapping shrimp [84]. Nonetheless, there are intriguing commonalities in the mechanistic underpinnings of queen and worker caste determination in Hymenoptera and solider caste differentiation in termites, including the involvement of juvenile hormone and storage proteins such as hexamerins [85]. Since termite workers are derived from juvenile stages, and in many species, can mature into neotenic reproductives or soldiers, the path of caste determination is very different (reviewed in [86]). In addition, hymenopteran workers are all female, while termite workers are both male and female (reviewed in [86]). Comparing the effects of DNA methylation on reproductive and solider caste determination in termites to effects in Hymenoptera could be extremely informative.

### 7.2. Mechanistic Understanding of DNA Methylation

In order to more fully understand the effects of methylation on caste determination, we need to better understand the effects of differential methylation on gene expression and splicing. There is growing knowledge on the precise locations within social insect genomes that are generally methylated relative to the beginning and end of transcription [33]. With more studies that directly compare the locations of methylated sites to splicing sites, we can better understand how alternative splicing may be regulated by DNA methylation. In addition, it would be valuable to know whether there are differences between consistent methylation and differential methylation in how and where genes are methylated. For example, are consistently methylated genes methylated more frequently in certain regions of genes, and how does this affect expression and splicing [33]?

Another avenue that could help us better understand the effect of DNA methylation on caste determination is understanding the dynamics of methylation patterns during development and during adulthood. How changeable are methylation patterns within an individual? Methylation changes may even be important for shorter-term plasticity, specifically, learning in adult worker honeybees [87]. In addition, we know that it is possible to reverse the effects of maternal care on methylation in adult mice [51]; what about caste-related methylation differences? Do methylation patterns change when workers reproduce under queenless conditions? If these patterns are changeable in adults, perhaps this stems from behavioral flexibility in solitary ancestors. Do solitary species that have laying and nonlaying periods undergo shifts in methylation? Such comparisons could provide new insight into the mechanistic regulation and evolution of castes.

 In conclusion, the study of epigenetic modifications in social insects has already provided useful and intriguing information about the mechanisms of caste determination in honeybees, as well as a better appreciation of the complexities of gene regulation. There is still a great deal of work to be done in this area related to mechanisms, evolution, and imprinting. Further research could provide valuable insights into not only the mechanisms, but also the evolutionary origins of eusociality.

## Figures and Tables

**Table 1:** Features of consistent and differential methylation in social insects.

Consistent methylation	Differential methylation
Sites consistently methylated	Methylation varies across tissues, castes, and individuals
Depleted CpG content [22, 23]	Less depleted CpG content [22, 23]
Primarily found in exons [33]	Primarily found in exons [33]
Consistent expression levels/splicing patterns across tissues and castes [22]	Variable expression levels/splicing patterns across tissues and castes [23]
Well-conserved across insect taxa [47]	Not yet known whether patterns conserved or divergent across taxa
